# Long non-coding RNA HOTAIR modulates intervertebral disc degenerative changes via Wnt/β-catenin pathway

**DOI:** 10.1186/s13075-019-1986-8

**Published:** 2019-09-03

**Authors:** Shengfeng Zhan, Kun Wang, Yu Song, Shuai Li, Huipeng Yin, Rongjin Luo, Zhiwei Liao, Xinghuo Wu, Yukun Zhang, Cao Yang

**Affiliations:** 1 Department of Orthopaedics, Union Hospital, Tongji Medical College, Huazhong University of Science and Technology, Wuhan, 430022 China; 2 Department of Orthopaedics, Enshi Center Hospital, Enshi, China

**Keywords:** Intervertebral disc degeneration, LncRNA HOTAIR, Wnt/β-catenin, Senescence, Apoptosis, Extracellular matrix

## Abstract

**Background:**

Intervertebral disc degeneration (IDD) has a complicated and enigmatic pathogenic process. Accumulating evidence shows that long non-coding RNAs (LncRNAs) play a role in the pathogenesis of IDD. This study aimed to investigate the expression and role of the LncRNA HOTAIR in IDD pathogenesis.

**Methods:**

Nucleus pulposus (NP) tissue samples from 10 patients with idiopathic scoliosis and 10 patients with lumbar disc herniation were collected. qRT-PCR was used to assess the expression of HOTAIR and ECM-related genes; western blotting was used to detect the expression of senescence biomarkers, apoptosis-related proteins, and Wnt/β-catenin pathway; flow cytometry was used to detect apoptosis; and the MTT assay was used to determine cell proliferation. Moreover, a classic needle-punctured rat tail model was used to investigate the role of HOTAIR in IDD in vivo.

**Results:**

The results showed that the expression of HOTAIR significantly increased during IDD progression. The overexpression of HOTAIR was found to induce nucleus pulposus (NP) cell senescence, apoptosis, and extracellular matrix (ECM) degradation. HOTAIR silencing by RNA interference in NP cells prevented interleukin-1β-induced NP cell senescence, apoptosis, and ECM degradation. Furthermore, we found that the Wnt/β-catenin pathway played a role in regulating HOTAIR to induce these changes in NP cells. Moreover, HOTAIR inhibition in a rat model effectively attenuated IDD symptoms in vivo.

**Conclusions:**

Our findings confirmed that HOTAIR promoted NP cell senescence, apoptosis, and ECM degradation via the activation of the Wnt/β-catenin pathway, while silencing HOTAIR attenuated this degeneration process, indicating a potential therapeutic target against IDD.

## Background

Lower back pain (LBP) affects approximately 70–80% of the population at some point during their lives [1] and is a major issue in terms of socioeconomic cost and health care expenditure [2]. LBP is often associated with intervertebral disc (IVD) degeneration (IDD) [3], but the pathological mechanisms of IDD are unclear. Although conservative treatment and surgical approaches are commonly used to treat IDD, the results are limited to reducing the severity of symptoms. There is currently no effective treatment to reverse the progression of IDD; recurrence after treatment is common [4]. Thus, a new therapeutic target for the IDD process is necessary.

Long non-coding RNAs (lncRNAs) were recently found to be abundant in the mammalian genome [5] and to participate in the regulation of key cellular processes including differentiation, proliferation, and apoptosis [6]. HOTAIR, a 2158-bp lncRNA found at the mammalian HOXC locus, binds to and targets the Polycomb Repressive Complex 2 (PRC2) complex at the HOXD locus [7, 8]. Accumulating studies have implicated HOTAIR in many diseases. For example, the knockdown of HOTAIR was shown to reverse the overexpression of matrix metalloproteinases (MMPs) and to decrease chondrocyte apoptosis in interleukin (IL)-1β-induced temporomandibular joint osteoarthritis [9]. The overexpression of HOTAIR was shown to increase cancer invasiveness [10]. HOTAIR regulates osteogenic differentiation and proliferation in non-traumatic osteonecrosis of femoral head via miR-17-5p [11]. Furthermore, HOTAIR expression was shown to be increased in the cardiac tissues of patients with congenital heart diseases [12]. However, the role of HOTAIR in the progression of IDD is unclear.

As reported in the literature, Wnt/β-catenin signaling plays a role in IDD progression [13]. In the current study, we hypothesized that HOTAIR plays an important role in IDD by modulating nucleus pulposus (NP) cell senescence, apoptosis, and extracellular matrix (ECM) degradation by regulating the Wnt/β-catenin pathway. We collected human NP tissue samples to determine their HOTAIR expression levels. In addition, we conducted in vitro experiments on human NP cells to investigate the role of HOTAIR in IL-1β-induced senescence, apoptosis, and ECM degradation, as well as the relationship between HOTAIR overexpression and the Wnt/β-catenin signaling pathway. Finally, we explored the role of HOTAIR in IDD in vivo using a rat model.

## Methods

### Patient tissue samples

The experimental protocols in this study were approved by the Ethics Committee of Tongji Medical College, Huazhong University of Science and Technology, China, and followed the guidelines of the Helsinki Declaration [14]. All participants provided written informed consent for sample collection before the experiments. Lumbar NP tissue samples were harvested from ten patients with idiopathic scoliosis undergoing deformity correction surgery as normal controls (*n* = 4 women and *n* = 6 men; mean age 20.7 years; range 16–30 years). The patients in this group had no history of chronic lower back or leg pain before the deformity correction surgery. Degenerative lumbar NP tissues samples were harvested from ten lumbar disc herniation patients (*n* = 5 women and *n* = 5 men; mean age: 33.6 years; range: 21–59 years) undergoing surgery in Union Hospital, Tongji Medical College, Huazhong University of Science and Technology.

### Isolation and culture of human NP cells

Human NP tissues obtained from ten participants with idiopathic scoliosis were isolated as described previously [15] and maintained in Dulbecco’s modified Eagle’s medium (DMEM)/F12 (Gibco, Waltham, MA, USA) containing 15% fetal bovine serum (FBS; Gibco), 1% penicillin-streptomycin, 0.05% fungizone, and 25 μg/ml ascorbate, and cultured at 37 °C in a humidified atmosphere with 5% CO_2_. When the NP cells reached approximately 80% confluence, they were detached by trypsinization and sub-cultured in culture flasks. No significant changes in the morphology of primary (passage 0) and later-passage (passage 2) cells were observed. Accordingly, we used the second-passage cells cultured in a monolayer for the following experiments. The untreated cultured NP cells were used as the control group.

### Rat-tail disc degeneration model

A total of ten male Sprague-Dawley rats (3-month-old) were obtained from the Experimental Animal Center of Tongji Medical College (Wuhan, China). All experiments on animals were performed following protocols approved by the Animal Experimentation Committee of Huazhong University of Science and Technology.

The rat coccygeal IVDs were divided into three groups: sham + vector (empty plasmid vector, control group), IDD + vector, and IDD + short interfering (si)RNA against HOTAIR (siHOTAIR, a plasmid encoding siHOTAIR). The rats were anesthetized by intraperitoneal administration of ketamine (90 mg/kg) and ketamine hydrochloride (10 mg/kg) and were then placed in a prone position. The target coccygeal IVDs (Co6–7, Co7–8, and Co8–9) were located by digital palpation and confirmed by a trial radiograph. Co7–8 was left undisturbed, while Co6–7 and Co8–9 were punctured percutaneously with a 21-gauge needle as described previously [16]. The needle was rotated in an axial direction by 360° twice and held for 30 s before removal [17]. Thereafter, 2-μl empty vector or vector containing siHOTAIR was slowly injected into the disc using a 31-gauge needle [18, 19].

### RNA extraction and quantitative real-time (qRT)-PCR

Total RNA was extracted from human NP cells and tissues using TRIzol reagent (Ambion, Foster City, CA, USA) according to the manufacturer’s instructions. The primers used for qRT-PCR are listed in Table 1. Total RNA was reverse transcribed using PrimeScript RT Master Mix (Takara Bio, Shiga, Japan) according to the manufacturer’s instructions. qRT-PCR was performed using the One Step SYBR PrimeScript RT-PCR Kit (Takara Bio) to quantify the RNA or mRNA expression levels of HOTAIR, MMP-3, MMP-9, MMP-10, type II collagen, aggrecan, Wnt1, and β-catenin. Target mRNA expression levels were normalized against GAPDH. The relative expression levels were computed using the 2^−ΔΔCt^ method.

**Table 1:** Sequences of primers used for quantitative real-time PCR

Gene	Forward (5–3′)	Reverse (5–3′)
HOTAIR	CATTCTGCCCTGATTTCCG	ATCCGTTCCATTCCACTGCG
MMP3	TTCCTTGGATTGGAGGTGAC	AGCCTGGAGAATGTGAGTGG
MMP9	CAGTCCACCCTTGTGCTCTTCCCTG	ATCTCTGCCACCCGAGTGTAACCA
MMP10	CAGGTTATCCAAGAGGCATCCATAC	TTAGGCTCAACTCCTGGAAAGTCAT
Type II collagen	TCCAGATGACCTTCCTACGC	GGTATGTTTCGTGCAGCCAT
Aggrecan	TGAGCGGCAGCACTTTGAC	TGAGTACAGGAGGCTTGAGG
Wnt1	GAATCGCCGCTGGAACTGTC	GCGGAGGTGATAGCGAAGATAAACG
β-catenin	CAAGTGGGTGGTATAGAGG	AGTCCATAGTGAAGGCGAAC
GAPDH	TCAAGAAGGTGGTGAAGCAGG	TCAAAGGTGGAGGAGTGGGT

### Plasmid transfection and RNA interference

NP cells were transfected with a plasmid encoding HOTAIR, or with an empty vector (negative control group), using Lipofectamine 2000 (Invitrogen, Carlsbad, CA, USA) according to the manufacturer’s instructions. After 24 h of transfection, the cells were treated with either IL-1β (10 ng/mL) or XAV-939 (a Wnt/β-catenin inhibitor; 10 μmol/L; Selleck, Houson, TX, USA) for a further 24 h. The cells were then harvested for subsequent experiments.

The siHOTAIR and scrambled control siRNA (siScr) were obtained from RiboBio (Guangzhou, China). NP cells were transfected using Lipofectamine 2000 (Invitrogen) and then treated with IL-1β (10 ng/mL) for 24 h.

### Western blotting

Proteins were extracted from NP cells using RIPA lysis buffer. Western blotting was carried out as described previously [20] with antibodies against the following proteins: cleaved caspase-3, B-cell lymphoma-2(Bcl-2), B-cell lymphoma-2 associated X (Bax), p16, and p53 (Abcam, Cambridge, UK). After initial incubation, the membrane was cultured with horseradish peroxidase (HRP)-conjugated goat anti-rabbit secondary antibody (Boster, Wuhan, China). The gray value ratio of target band to the reference band reflected the relative protein levels with GAPDH as the internal reference protein.

### MTT assay

A 3-(4,5-dimethylthiazol-2-yl)-2,5-diphenyltetrazolium bromide (MTT) assay was carried out to determine NP cell numbers as an indicator of cell proliferation. Briefly, 3 × 10^3^NP cells/well were plated on 96-well plates. After plasmid transfection, 10 μl MTT (Sigma Aldrich, St. Louis, MO, USA) solution was added to each well, and NP cells were incubated for 4 h at 37 °C. Then, 150 μl dimethyl sulfoxide (Sigma Aldrich) was added to solubilize the formazan. Absorbance of samples was recorded at 568 nm.

### Flow cytometry

When the NP cells reached the logarithmic growth phase, 5 × 10^5^ cells/well were seeded onto 6-well plates. Following treatment with or without transfection for 48 h as described above, 0.25% trypsin (without EDTA) was used to digest the cells. Cell apoptosis was detected by flow cytometry using the Annexin V-APC/7-AAD Apoptosis Detection kit (KeyGEN, Nanjing, China) according to the manufacturer’s instructions.

NP cells were collected as described above. After treatment with or without transfection for 48 h as described above, the cells were digested with 0.25% trypsin (without EDTA) and fixed in ice-cold 70% ethanol overnight at 4 °C. Next, the cells were incubated with RNase (50 μg/ml, KeyGEN) for 30 min at 37 °C, followed by propidium iodide dye (50 μg/ml, KeyGEN) for a further 30 min. The cells were then analyzed using flow cytometry. The proportion of NP cells in each cell cycle phase was evaluated.

### Immunohistochemistry

NP samples were fixed in 10% formaldehyde for 24 h and embedded in paraffin. Then, the samples were sliced into 4-μm sections. Immunohistochemistry was carried out as described previously [20]. The sections were incubated with antibodies against Bax (1:100; Proteintech Group, Rosemont, IL, USA), Bcl-2 (1:100; Abcam), p16 (1:200; Thermo Fisher Scientific, Waltham, MA, USA), and p53 (1:100; Proteintech Group). Staining was performed using the Dako REAL EnVision Detection System, Peroxidase/DAB+, Rabbit/Mouse (Dako Cytomation, Glostrup, Denmark), according to the manufacturer’s instructions. The sections were imaged by microscopy (Olympus, Tokyo, Japan).

NP cell immunostaining of nuclear β-catenin was performed as follows. After incubation with different test substances, NP cells were washed with PBS and fixed with 4% buffered paraformaldehyde. The cells were then permeabilized with 0.5% Triton X-100 for 20 min at room temperature. Then, the recuperation of epitopes was performed, with inactivation of endogenous peroxidase using 3% hydrogen peroxide. Non-specific binding was blocked by incubation for 30 min with goat serum. The NP cells were then incubated with the primary antibody anti-β-catenin (1:100; Proteintech Group) overnight at 4 °C. After washing with PBS, the samples were incubated with secondary antibody and stained with Harris hematoxylin. Images were acquired using a microscope (Olympus).

### Immunofluorescence staining

Cultured NP cells were rinsed three times with PBS and fixed with 4% paraformaldehyde. After washing again with PBS, the cells were permeabilized with 0.5% Triton X-100 in PBS for 20 min and blocked with 5% FBS for 30 min. Thereafter, the NP cells were incubated overnight at 4 °C with antibodies against MMP-9 (1:100; Abcam) and then incubated with a Cy3-conjugated goat anti-rabbit IgG antibody (1:100; Boster Bio, Pleasanton, CA, USA) for 1 h at 37 °C. After washing in the dark, the cells were incubated with DAPI for 5 min. Cells were imaged using a fluorescence microscope (Olympus).

### Magnetic resonance imaging (MRI)

The effect of siHOTAIR treatment on coccygeal intervertebral disc degeneration was evaluated using a 7.0 T MR scanner (Bruker BioSpec70/20USR, Bruker, Germany) at 4 weeks post-surgery. The rats were placed in a prone position, and their tails were straightened in the MR scanner. Serial T2-weighted sagittal sections were obtained using the following settings: fast spin echo sequence with time to repetition of 2000 ms and time to echo of 36 ms; flip angle = 180; field of view = 60 × 30 mm; matrix = 256; number of excitations = 8; slice thickness = 0.8 mm. The rat tail and spine MRI sections were analyzed according to the classification method of Pfirrmann et al. [21].

### Radiographic analysis

Radiographs of the rat tails were taken in an X-ray system (DRX-Evolution, Carestream Health, China) before surgery and 4 weeks post-surgery. The rats were placed in a prone position, and their tails were straightened. IVD heights were measured using digital radiographs and Image J software. IVD height was expressed based on the disc height index (DHI), as described previously [22].

### Histological evaluation

Rats were euthanized at 4 weeks post-surgery. Whole discs with adjacent vertebrae were dissected and removed. The specimens were fixed in 10% formalin and decalcified in 10% EDTA for 30 days. Then, the specimens were embedded in paraffin blocks and sliced into 5-μm sections with a microtome (Leica RM2145). Subsequently, the sections were stained with hematoxylin and eosin (H&E) to obtain histological scores. The slides were evaluated in a blind fashion and graded using a previously established definition [23].

### Statistical analysis

The results are presented as the mean ± standard deviation (SD) based on at least three independent experiments. Statistical analysis was performed using SPSS21.0 (IBM, Armonk, NY, USA). The differences between groups were analyzed using Student’s *t* test or one-way analysis of variance (ANOVA). A value of *P* < 0.05 was considered statistically significant.

## Results

### HOTAIR expression is elevated in human NP tissues from IDD patients

**Fig. 1:**
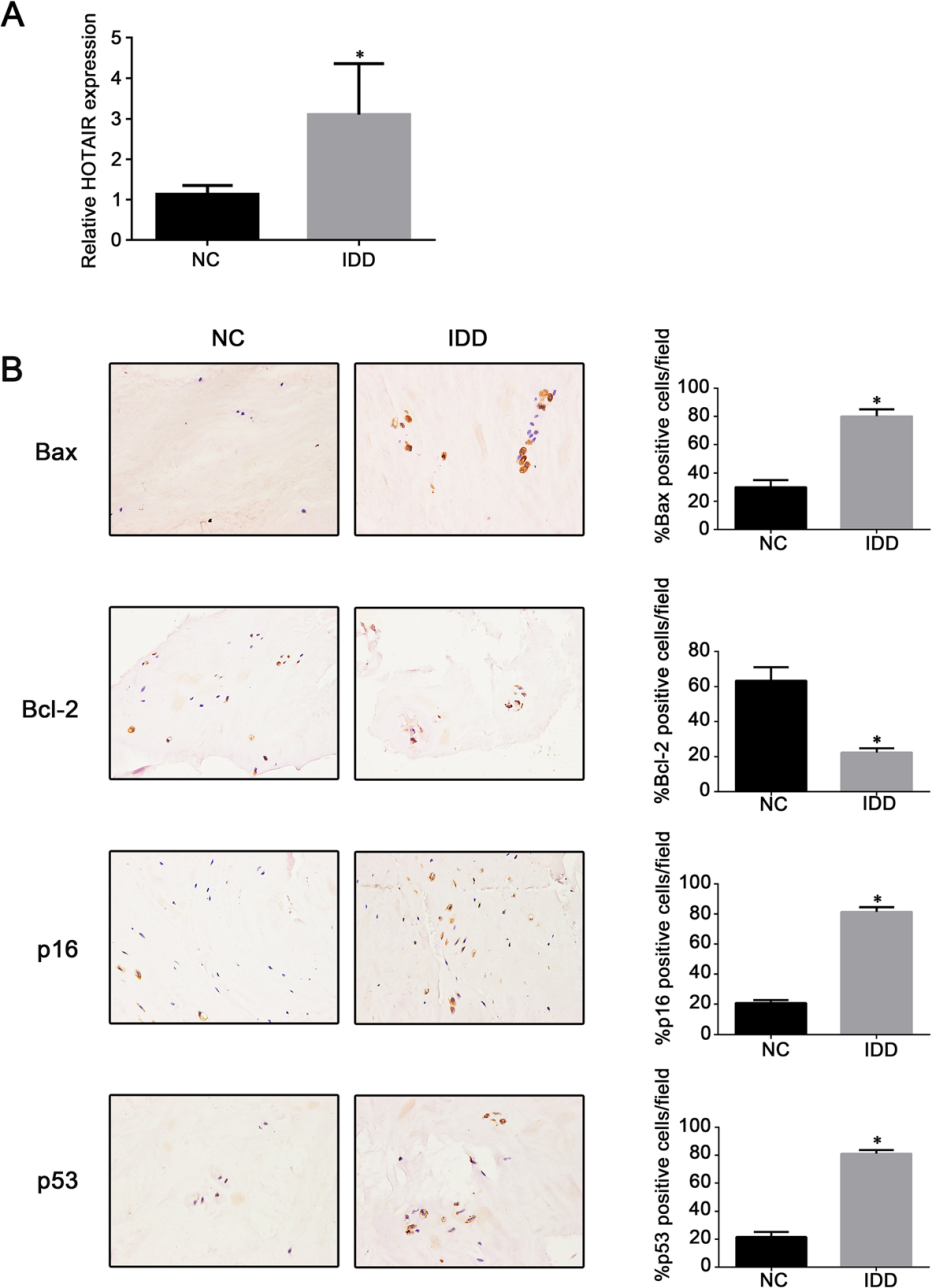
HOTAIR expression in human nucleus pulposus (NP) tissues. **a** Expression of HOTAIR in 20 human NP tissues was determined by qRT-PCR. **b** Immunohistochemistry analysis of apoptosis-related molecules (Bax and Bcl-2) and senescence markers (p16 and p53). Magnification × 200. Data are presented as mean ± SD from 3 samples for each group in 3 independent experiments. Data were analyzed using Student’s *t* test. *P < 0.05 vs. normal control (NC) group

To study the role of HOTAIR in the pathogenesis of IDD, we measured the expression of HOTAIR in NP tissues from idiopathic scoliosis patients or IDD patients using qRT-PCR. The results showed that the expression of HOTAIR was significantly upregulated in IDD patients compared to that in idiopathic scoliosis patients (Fig. 1a). Furthermore, immunohistochemistry showed an increase in the proportion of Bax-, p16-, and p53-positive cells, as well as a decrease in the proportion of Bcl-2-positive cells in the IDD group compared to that in the control group (Fig. 1b).

### HOTAIR expression in NP cells in response to IL-1β stimulation

**Fig. 2:**
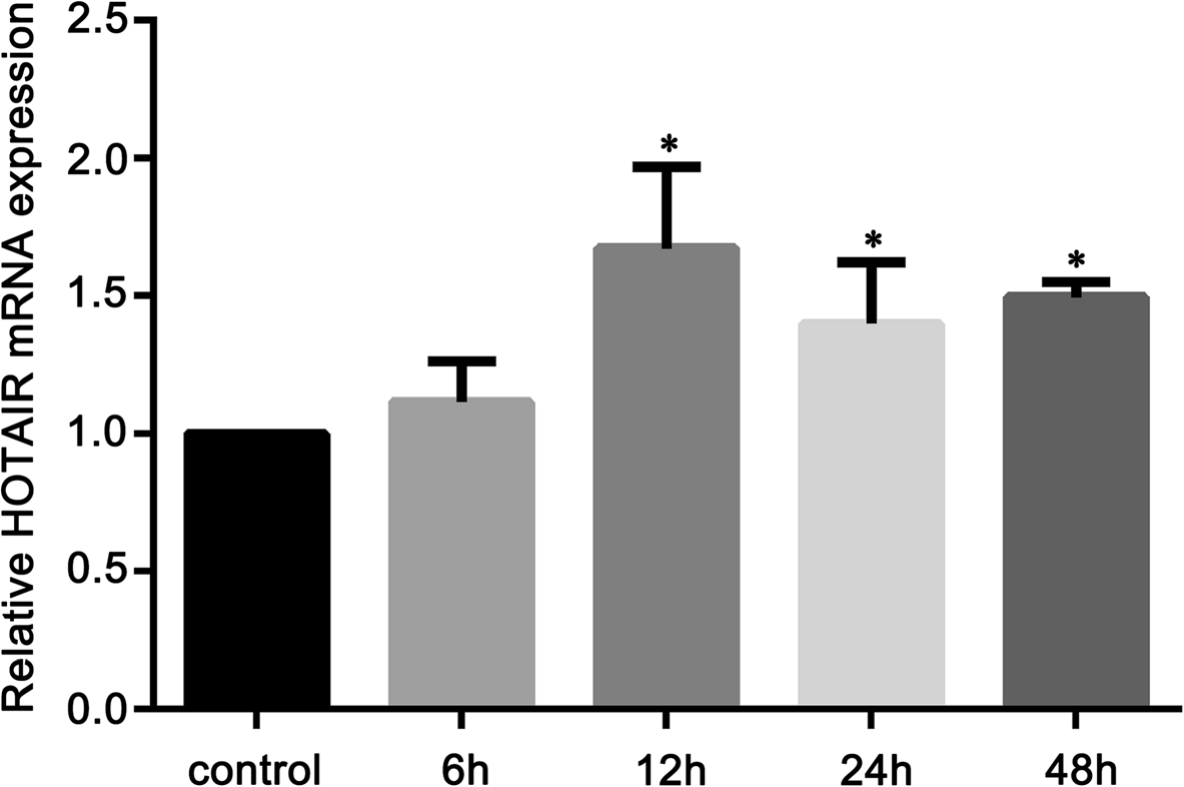
Interleukin (IL)-1β induces HOTAIR expression in NP cells. HOTAIR expression levels were determined by qRT-PCR in NP cells treated with IL-1β (10 ng/mL) for 6, 12, 24, or 48 h. Untreated NP cells were used as the control group. Data are presented as mean ± SD from 3 samples for each group in 3 independent experiments. Data were analyzed using one-way analysis of variance followed by a Tukey test. *P < 0.05 vs. control group

Previous studies have reported the role of IL-1β in inducing IDD. To analyze the expression of HOTAIR in the progression of IDD, NP cells were treated with IL-1β (10 ng/mL) for 6, 12, 24, or 48 h, and the expression of HOTAIR was then measured using qRT-PCR. As shown in Fig. 2, there were no significant changes in HOTAIR expression after 6 h of IL-1β treatment compared to that in the control group. However, IL-1β treatment significantly upregulated the expression of HOTAIR at 12, 24, and 48 h compared to that in the control group. Furthermore, the expression of HOTAIR was not significantly different at 12, 24, or 48 h.

### Overexpression of HOTAIR induces NP cell senescence, apoptosis, and ECM degradation

**Fig. 3:**
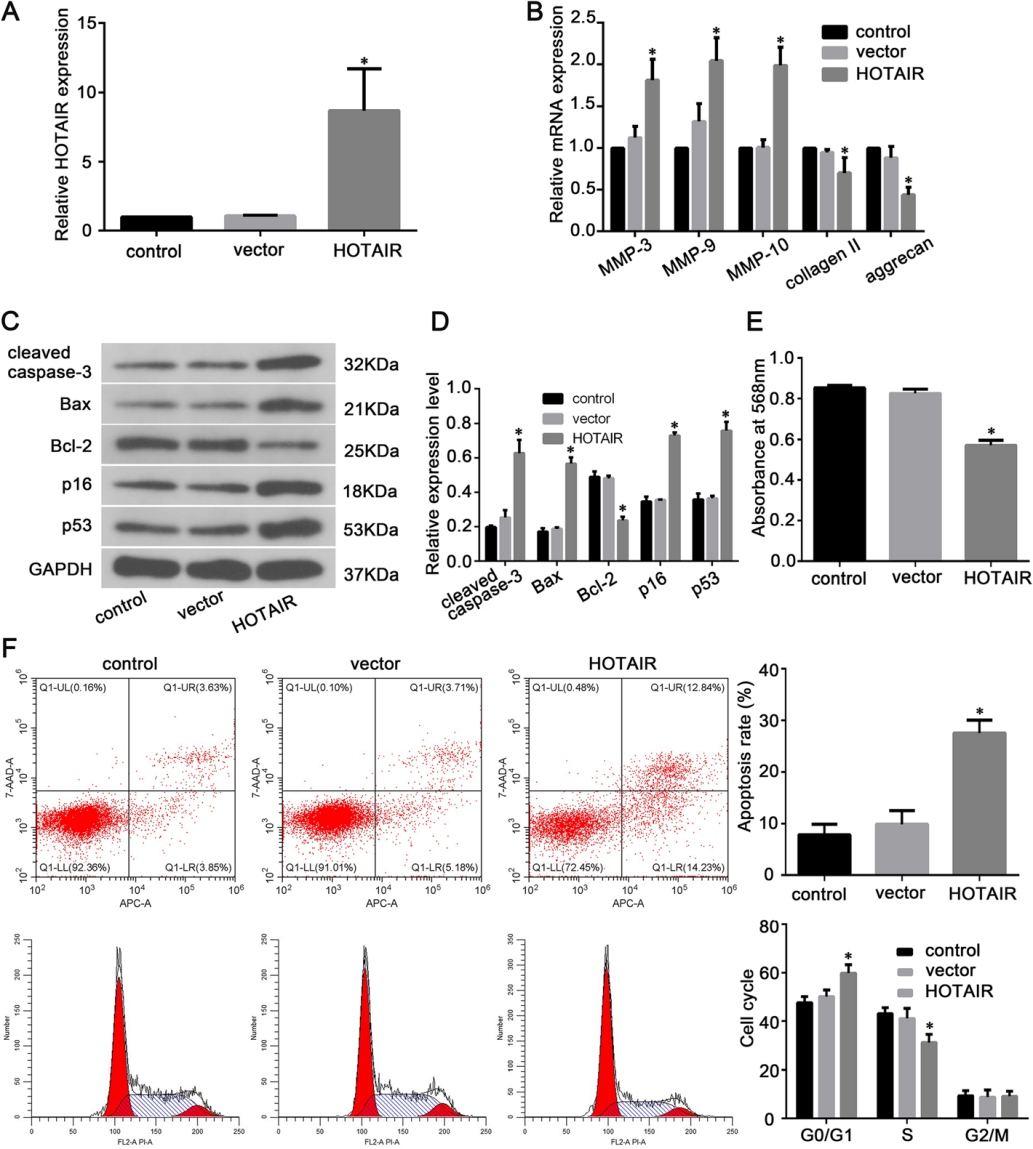
HOTAIR enhances human NP cell senescence, apoptosis, and ECM degradation. **a** HOTAIR expression in NP cells was determined by RT-qPCR following transfection. **b** RT-qPCR analysis of matrix metalloproteinase (MMP)-3, MMP-9, MMP-10, type II collagen, and aggrecan mRNA expression in NP cells following transfection. **c** Western blot images of protein expression (cleaved caspase-3, Bcl-2, Bax, p16, and p53) in NP cells. **d** Histogram showing the relative protein expression levels of cleaved caspase-3, Bcl-2, Bax, p16, and p53 in NP cells. **e** Proliferation of NP cells was determined by an MTT assay. **f** Flow cytometric distributions of apoptosis and cell cycle phases of NP cells. Data are presented as mean ± SD from 3 samples for each group in 3 independent experiments. Data were analyzed using Student’s *t*-test. *P < 0.05 vs. vector group

To explore the potential role of HOTAIR in NP cell senescence, apoptosis, and ECM degradation during IDD, we induced the overexpression of HOTAIR in NP cells. As shown in Fig. 3a, HOTAIR expression was markedly elevated after transfection with a plasmid encoding HOTAIR, while empty vector-treated NP cells showed no significant changes in HOTAIR expression compared to that in the control group. The mRNA expression levels of MMP-3, MMP-9, MMP-10, type II collagen, and aggrecan were also measured by qRT-PCR, and the protein expression of cleaved caspase-3, Bcl-2, Bax, p16, and p53 was measured by western blotting. The overexpression of HOTAIR led to the upregulation of cleaved caspase-3, p16, p53, Bax, MMP-3, MMP-9, and MMP-10 expression, as well as the downregulation of Bcl-2, type II collagen, and aggrecan expression (Fig. 3b–d). We further examined the effect of HOTAIR on the proliferation of NP cells using an MTT assay. HOTAIR overexpression significantly reduced the viability of NP cells at 48 h post-transfection compared to that of empty vector-treated cells and untreated cells (Fig. 3e). Flow cytometry analysis also confirmed that NP cell apoptotic rates were significantly increased after 48 h in HOTAIR-overexpressed cells compared to those of control cells; additionally, HOTAIR overexpression significantly reduced the proportion of S-phase cells, but increased the proportion of G0/G1-phase cells (Fig. 3f).

### Silencing of HOTAIR attenuates IL-1β-induced NP cell senescence, apoptosis, and ECM degradation

**Fig. 4:**
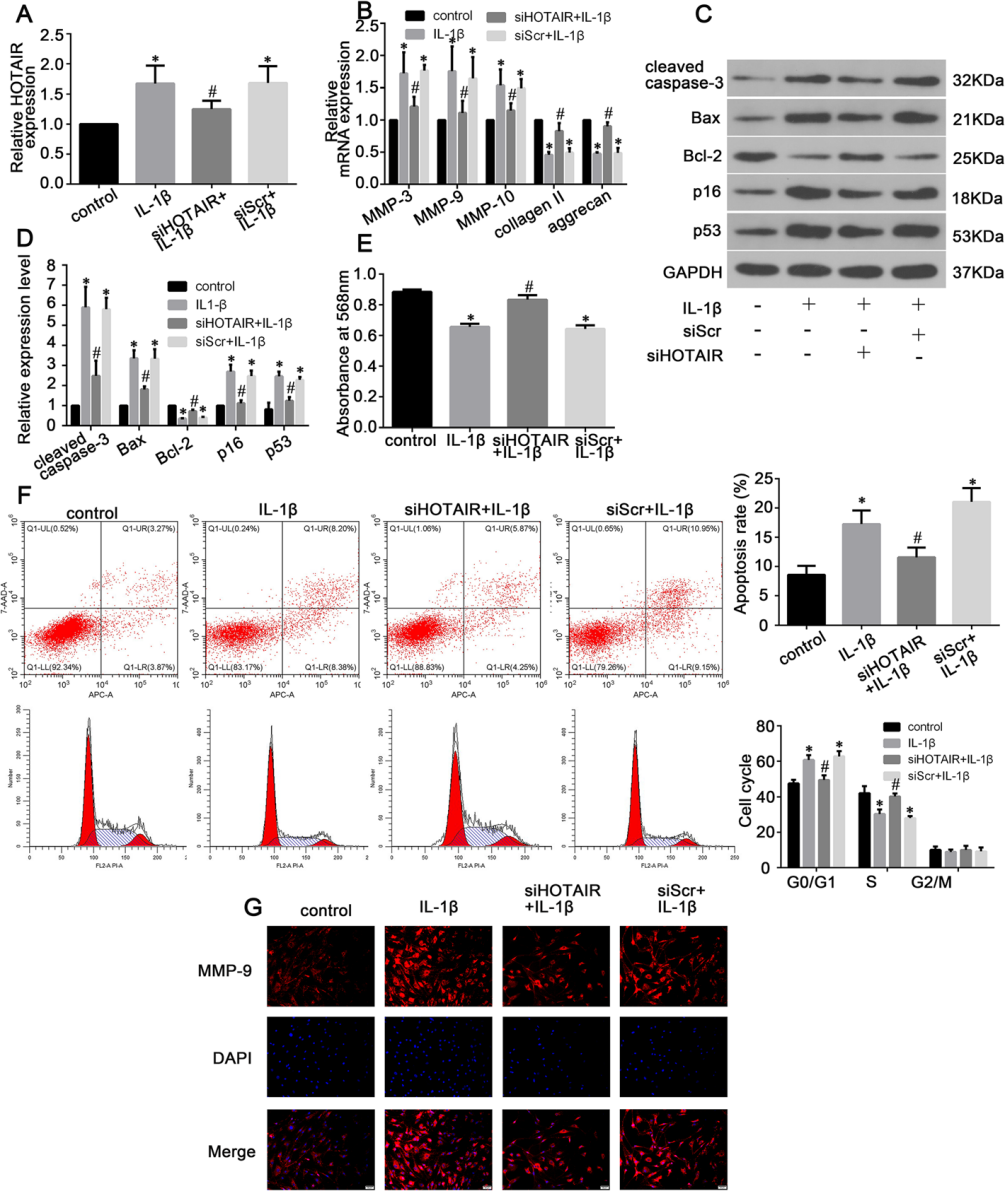
Knockdown of HOTAIR attenuates IL-1β-induced human NP cell senescence, apoptosis, and ECM degradation. **a** RT-qPCR analysis of HOTAIR expression in NP cells. **b** RT-qPCR analysis of MMP-3, MMP-9, MMP-10, type II collagen, and aggrecan expression in NP cells. **c** Protein expression levels (cleaved caspase-3, Bcl-2, Bax, p16, and p53) as analyzed by western blot. **d** Histogram showing the relative protein expression levels of cleaved caspase-3, Bax, p16, and p53 in the four groups. **e** An MTT assay was performed to measure the proliferation of NP cells following plasmid transfection and IL-1β treatment. **f** Flow cytometric distributions of apoptosis and cell cycle phases of NP cells after plasmid transfection and IL-1β treatment. **g** Immunofluorescence staining of MMP-9 in NP cells from different groups. Data are presented as mean ± SD from 3 samples for each group in 3 independent experiments. Data were analyzed using Student’s *t*-test. *P < 0.05 vs. control group. ^#^*P* < 0.05 vs. siScr + IL-1β group

To further investigate whether HOTAIR plays a role in IL-1β-induced NP cell senescence, apoptosis, and ECM degradation, NP cells were transfected with siHOTAIR to silence HOTAIR expression. The results showed that HOTAIR was effectively suppressed in siHOTAIR-transfected NP cells (Fig. 4a). Moreover, we treated the transfected NP cells with IL-1β (10 ng/mL) for 24 h. The mRNA expression of the ECM-related catabolic enzymes MMP-3, MMP-9, and MMP-10 was increased by IL-1β stimulation, and IL-1β clearly decreased the mRNA expression of type II collagen and aggrecan (Fig. 4b). Treatment with siHOTAIR markedly attenuated these IL-1β-induced changes (Fig. 4b). Furthermore, IL-1β treatment markedly increased cleaved caspase-3, Bax, p16, and p53 protein expression (Fig. 4c and d). This change was also attenuated by siHOTAIR transfection (Fig. 4c and d). Cell proliferation declined in NP cells treated with IL-1β, whereas siHOTAIR significantly rescued this reduction in viability, as shown by MTT assays (Fig. 4e). The rates of NP cell apoptosis, as well as the cell cycle stages of NP cells, were then assessed by flow cytometry. IL-1β significantly upregulated NP cell apoptotic rates and increased the proportion of G0/G1-phase cells. Treatment with siHOTAIR significantly attenuated NP cell apoptotic rates and decreased the number of cells at the G0/G1 phase (Fig. 4f). Furthermore, immunofluorescence staining for MMP-9 expression was performed in NP cells after plasmid transfection and IL-1β treatment and showed that MMP-9 expression was increased in IL-1β-stimulated NP cells, but that siHOTAIR attenuated this IL-1β-induced increase in MMP-9 expression (Fig. 4g).

### Effect of HOTAIR overexpression on Wnt/β-catenin signaling in human NP cells

**Fig. 5:**
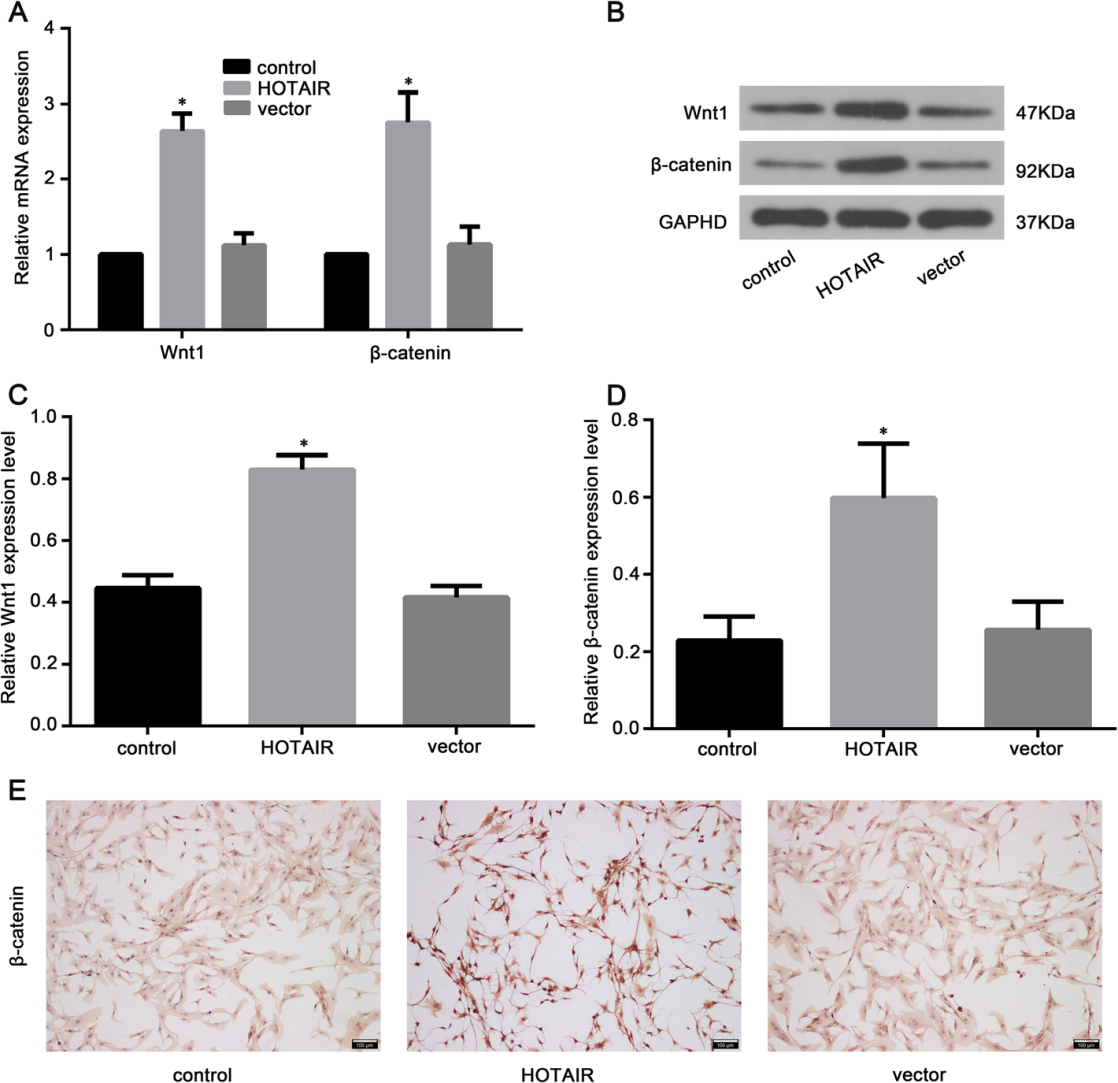
HOTAIR activates the Wnt/β-catenin pathway in human NP cells. **a** The mRNA expression of Wnt1 and β-catenin in NP cells was analyzed by qRT-PCR. **b**–**d** The protein expression levels of Wnt1 and β-catenin in NP cells were analyzed by western blot. **e** β-catenin expression was determined by immunohistochemistry staining in NP cells. Scale bar = 100 μm. Data are presented as mean ± SD from 3 samples for each group in 3 independent experiments. Data were analyzed using Student’s *t* test. *P < 0.05 vs. vector group

Previous studies have reported that the Wnt/β-catenin signaling pathway plays a major role in the pathogenesis of IDD [13]. Therefore, we hypothesized that HOTAIR expression promotes NP cell senescence, apoptosis, and ECM degradation during IDD progression through the regulation of Wnt/β-catenin signaling. Through qRT-PCR, we found that Wnt1 and β-catenin mRNA expression in NP cells overexpressing HOTAIR showed a marked increase compared to that in cells in the control group (Fig. 5a). In addition, we measured the Wnt1 and β-catenin protein expression in cells overexpressing HOTAIR by western blotting. As shown in Fig. 5b–d, NP cells overexpressing HOTAIR showed a marked upregulation of Wnt1 and β-catenin expression. Furthermore, immunohistochemistry was performed and showed accumulation of nuclear β-catenin in NP cells after treatment with HOTAIR, indicating active Wnt signaling (Fig. 5e).

### HOTAIR promoted NP cell senescence, apoptosis, and ECM degradation via the Wnt/β-catenin signaling pathway

**Fig. 6:**
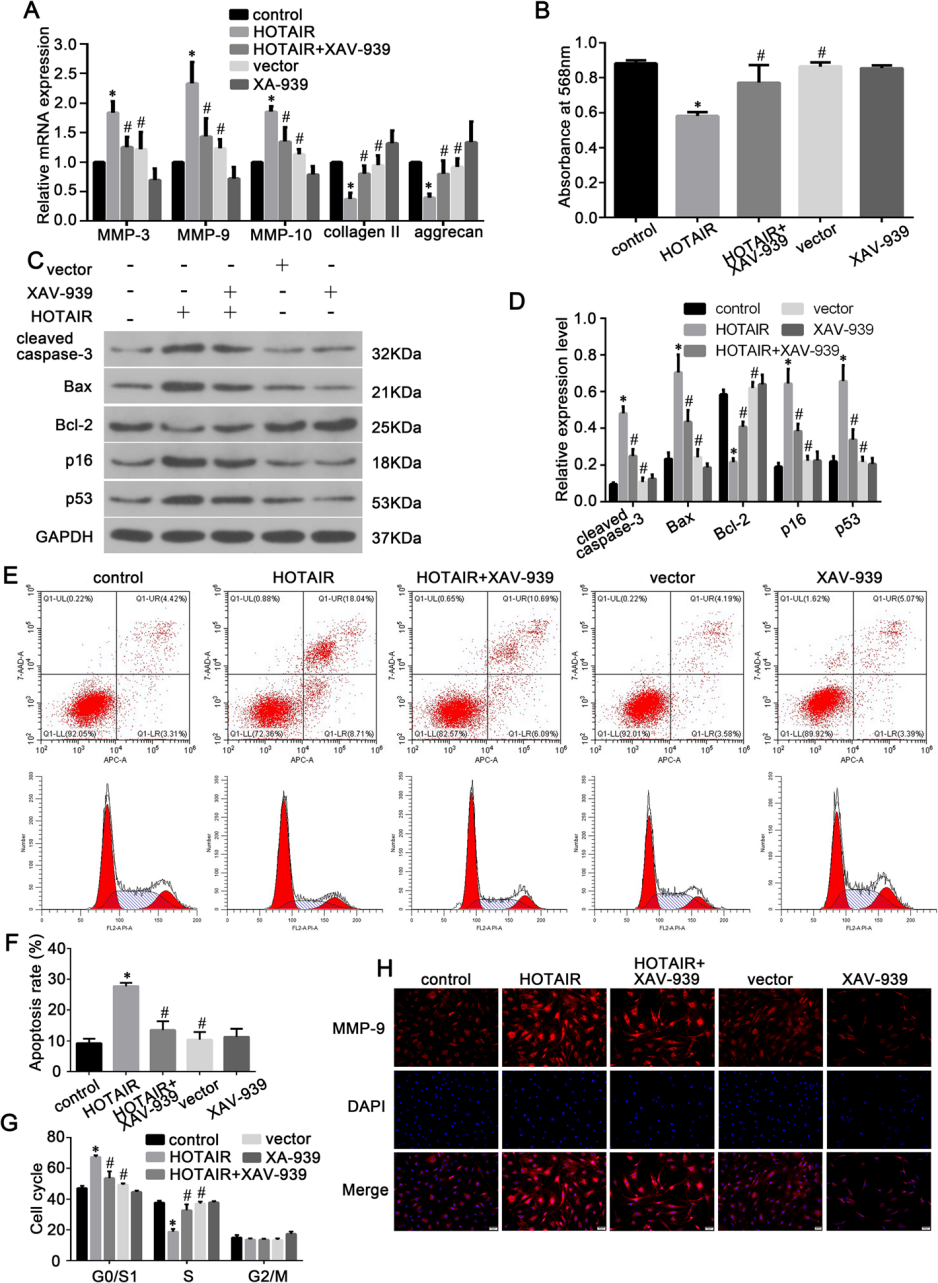
HOTAIR promotes human NP cell senescence, apoptosis, and ECM degradation via the Wnt/β-catenin pathway. **a** RT-qPCR analysis of ECM catabolic enzymes (MMP-3, MMP-9, and MMP-10), type II collagen, and aggrecan expression in NP cells. **b** An MTT assay was performed to determine the proliferation of NP cells. **c** Western blot analysis of protein expression levels (cleaved caspase-3, Bcl-2, Bax, p16, and p53). **d** Histogram showing the relative protein expression levels of cleaved caspase-3, Bax, Bcl-2, p16, and p53 in the five groups. **e**–**g** Flow cytometric distributions of apoptosis and cell cycle phases of NP cells after plasmid transfection and XAV-939 treatment. **h** Immunofluorescence staining of MMP-9 in NP cells from different groups. Data are presented as mean ± SD from 3 samples for each group in 3 independent experiments. Data were analyzed using Student’s *t* test. *P < 0.05 vs. control group. ^#^*P* < 0.05 vs. HOTAIR group

To further explore the potential role of the Wnt/β-catenin pathway in the promotion of NP cell senescence, apoptosis, and ECM degradation by HOTAIR, NP cells were stimulated by inducing the overexpression of HOTAIR or the overexpression of HOTAIR and XAV-939(an inhibitor of Wnt/β-catenin signaling). As shown in Fig. 6a, HOTAIR overexpression substantially increased the mRNA expression of MMP-3, MMP-9, and MMP-10 and notably decreased type II collagen and aggrecan mRNA expression. Furthermore, XAV-939 significantly attenuated these changes in expression (*P* < 0.05). An MTT assay showed that HOTAIR overexpression reduced the proliferation of NP cells and that XAV-939 significantly rescued this reduction (Fig. 6b). Additionally, the overexpression of HOTAIR upregulated the protein expression of cleaved caspase-3, Bax, p16, and p53 and downregulated the protein expression of Bcl-2; these changes were reversed by XAV-939 (Fig. 6c and d). Treatment with XAV-939 significantly attenuated the HOTAIR-induced changes in cell apoptotic rates and cell cycle progression in human NP cells (Fig. 6e–g). Consistent with these results, immunofluorescence staining showed similar changes in MMP-9 expression in NP cells under different treatment conditions (Fig. 6h). These data suggest that HOTAIR promotes NP senescence, apoptosis, and ECM degradation through the Wnt/β-catenin pathway.

### siHOTAIR partially reverses IDD progression in vivo

**Fig. 7:**
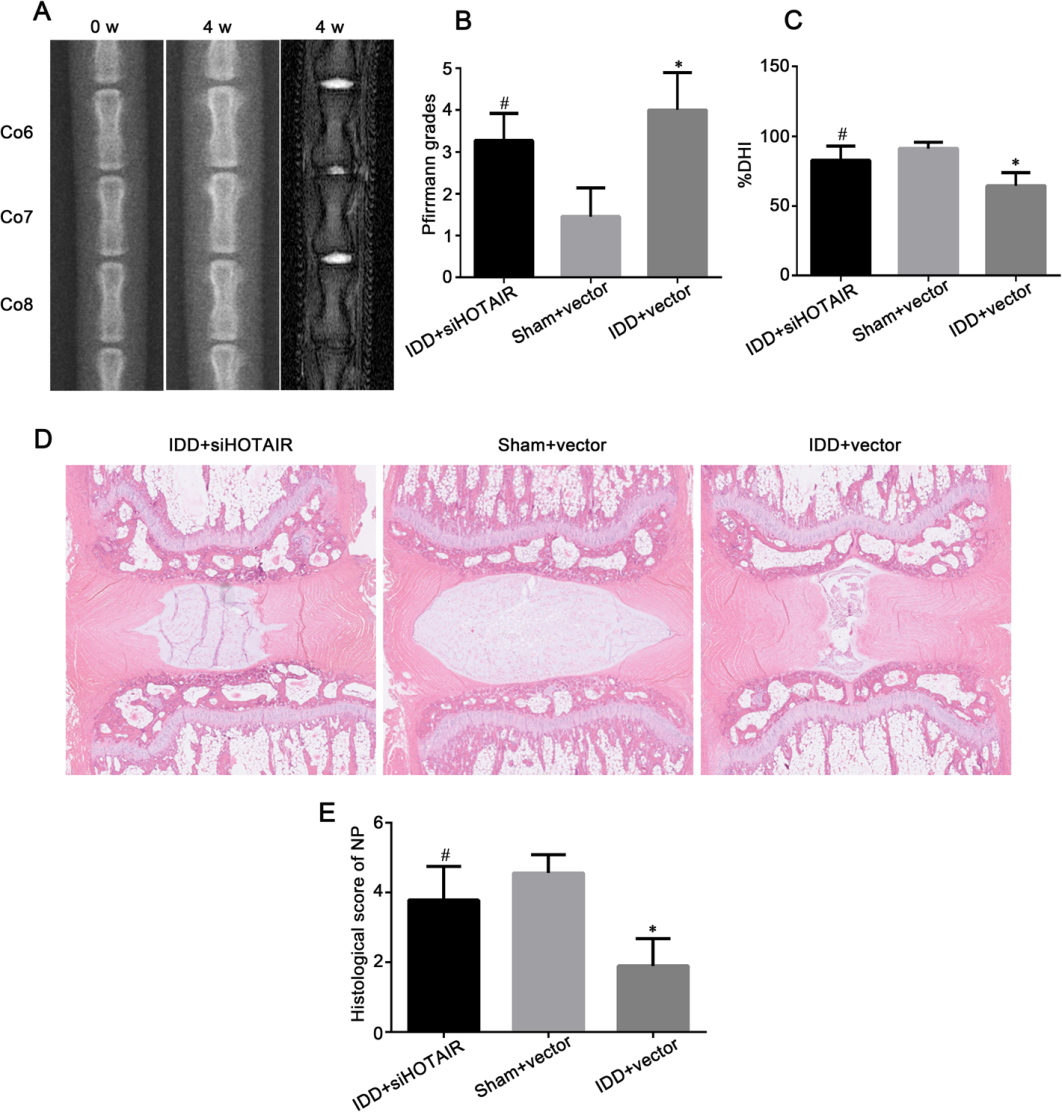
siHOTAIR partially prevents intervertebral disc degeneration (IDD) in vivo. **a** Radiographic images and representative T2-weighted MR sagittal images of rat tails at 4 weeks post-surgery. **b** Pfirrmann MRI grade scores of three groups at 4 weeks post-surgery. **c** The disc height index (DHI) was calculated at 4 weeks post-surgery. **d** Representative H&E staining of disc samples from different experimental groups at 4 weeks post-surgery. Magnification × 40. **e** Histological scores at 4 weeks post-surgery in three groups. Data are presented as mean ± SD from at least 3 samples for each group in 3 independent experiments. Data were analyzed using Student’s *t* test. *P < 0.05 vs. Sham + vector group. ^#^*P* < 0.05 vs. IDD + vector group

To investigate the role of HOTAIR in vivo, we performed a disc puncture procedure to model IDD in rats, which is a well-established method. As shown in Fig. 7a and b, MRI revealed degenerative changes in IVDs and altered structures of the coccygeal intervertebral discs after surgery. The MRI grading of IDD + vector group discs was compared with that of the IDD + siHOTAIR group discs. The IDD + vector group discs showed significantly more degeneration than the IDD + siHOTAIR group discs. Radiographic assessment of disc height was carried out to determine the DHI, which was calculated before and after surgery. As shown in Fig. 7a and c, empty vector injection resulted in a significant decrease in %DHI compared to siHOTAIR injection in degenerative discs. H&E staining showed a range of morphological changes after surgery (Fig. 7d). There was a significant difference in the histological scores between the IDD + vector group and the IDD + siHOTAIR group (Fig. 7e).

## Discussion

An increasing body of evidence suggests that the upregulation HOTAIR expression plays a role in the pathology of multiple diseases. For example, Huet al. found that HOTAIR promotes osteoarthritis progression through miR-17-5p/FUT2 signaling [24]. Moreover, it was recently reported that HOTAIR influences cell metastasis and apoptosis through miR-20a-5p/HMGA2 signaling in breast cancer [25]. Additionally, Hong et al. found that HOTAIR promotes renal cell carcinoma tumorigenesis through the miR-217/HIF-1α/AXL axis [26]. In the current study, we demonstrated a clear increase in HOTAIR expression in degenerative NP tissues and cells. Moreover, our results indicated that HOTAIR upregulation played an important role in the development of IDD.

Previous reports have demonstrated the role of IL-1β in inducing apoptosis and senescence in IVD cells [27, 28]. Furthermore, the overexpression of Bcl-2 in IVD cells has been found to effectively prevent cell apoptosis and reduce the expression of caspase 3 [29]. In contrast, the overexpression of HOTAIR is known to lead to adverse results. In this study, the expression of Bcl-2 was decreased, while the expression of Bax and cleaved caspase-3 was increased in HOTAIR-overexpressing NP cells compared to that in control NP cells; these results were consistent with previous findings [30]. Similarly, reduced cell proliferation was observed when HOTAIR overexpression was induced in NP cells, as shown in the NP cells treated with a Wnt/β-catenin activator [31]. In addition, the senescence of NP cells is a common feature of IDD [32]. The current study revealed that HOTAIR potentially upregulated senescence biomarkers (p16 and p53) and increased the proportion of cells in G0/G1 cell cycle arrest in degenerated NP cells compared to that in normal NP cells, consistent with previous studies [32, 33].

Moreover, NP cells in the IVD play a significant role in maintaining ECM homeostasis [34]. The NP ECM mainly consists of proteoglycans (primarily aggrecan) and type II collagen, which maintain the physiological functions of the IVD [34, 35]. Previous studies have demonstrated that MMPs are important enzymes for the cleavage of collagen and aggrecan in the NP ECM; the upregulation of MMPs is known to cause ECM degradation and IDD progression [36]. Decreased ECM function, increased degradative enzyme production, and increased inflammatory cytokine expression contribute to a weakened structural integrity and accelerate IVD degeneration [37]. In IDD, MMP-3 is highly expressed and reduces the expression of both type II collagen and proteoglycans [38]. MMP-9 expression is strongly associated with disc damage, and increased MMP-9 expression is known to exacerbate elastin degradation [39, 40]. MMP-10 expression has a strong correlation with IL-1 and can activate other members of the MMP family such as proMMP-1, proMMP-8, and proMMP-13 [41, 42]. The inflammatory cytokine IL-1β is significantly upregulated in IDD and increases the expression of ECM-degrading enzymes [43]. In this study, IL-1β expression was induced to cause normal NP cells to mimic the pathophysiology of IDD in vitro. Predictably, we found that stimulation by IL-1β induced the upregulation of MMP-3, MMP-9, and MMP-10, as well as the downregulation of type II collagen and aggrecan. Furthermore, the overexpression of HOTAIR induced the expression of MMPs in NP cells, consistent with the effects of IL-1β stimulation. Moreover, the inhibition of HOTAIR expression reversed the effects attributed to IL-1β stimulation. These results suggested that HOTAIR is a potent activator of IDD progression.

Previous findings have revealed that HOTAIR can decrease the expression of Wnt inhibitory factor 1, as well as activate the Wnt/β-catenin signaling pathway [44]. In this study, the expression levels of Wnt1 and β-catenin in NP cells overexpressing HOTAIR were significantly higher than those in normal NP cells. As previous studies have demonstrated, the Wnt/β-catenin signaling pathway plays a regulatory function in IDD [31]. Hiyama et al. found that activating the Wnt/β-catenin signaling pathway enhanced IVD cell senescence and apoptosis [45], consistent with our findings. Our study also showed that the expression of MMP-3, MMP-9, and MMP-10 was elevated following the activation of the Wnt/β-catenin pathway, consistent with previous studies [45]. Moreover, NP cells overexpressing HOTAIR and transfected with XAV-939 showed a significant inhibition of the Wnt/β-catenin signal pathway as well as reduced rates of senescence and apoptosis. These results suggested that HOTAIR may have a significant impact on the progression of IDD by activating the Wnt/β-catenin pathway.

In this study, we investigated the effects of HOTAIR on IDD using a rat IDD model. Rats in which HOTAIR expression was suppressed showed significant decreases in MRI grading, but increases in %DHI and morphological scores. Therefore, the results of our in vivo studies support the results of our in vitro analyses, indicating that HOTAIR promoted IDD.

The present study had a major limitation. All in vitro experiments were performed using normal NP cells obtained from scoliosis patients. It is possible that an underlying genetic condition exists in the NP tissues of scoliosis patients, which could influence our results. Therefore, it is necessary to perform further studies using NP cells from scoliosis patients and healthy patients to address this issue.

## Conclusion

To sum up, our findings suggest that HOTAIR plays an important role in IDD. The overexpression of HOTAIR was found to enhance NP cell senescence, apoptosis, and ECM degradation by activating the Wnt/β-catenin pathway in IDD. Hence, the development of specific inhibitors against HOTAIR to effectively decrease or control HOTAIR expression may be beneficial for the treatment of IDD.

## Data Availability

Data sharing is not applicable to this article as no datasets were generated or analyzed during the current study.

## Abbreviations

IVD: Intervertebral disc
IDD: Intervertebral disc degeneration
LncRNA: Long non-coding RNA
NP: Nucleus pulposus
ECM: Extracellular matrix
LBP: Lower back pain
MMP: Matrix metalloproteinase
IL: Interleukin
SD: Standard deviation
siRNA: Short interfering RNA
siHOTAIR: Short interfering RNA against HOTAIR
siScr: Scrambled control siRNA
MRI: Magnetic resonance imaging
MTT: 3-(4,5-Dimethylthiazol-2-yl)-2,5-diphenyltetrazolium bromide
DHI: Disc height index
Bax: B cell lymphoma-2 associated X
Bcl-2: B cell lymphoma-2
H&E: Hematoxylin and eosin
